# Organic solvents and MS susceptibility: Interaction with MS risk HLA genes

**DOI:** 10.1212/WNL.0000000000005906

**Published:** 2018-07-31

**Authors:** Anna Karin Hedström, Ola Hössjer, Michail Katsoulis, Ingrid Kockum, Tomas Olsson, Lars Alfredsson

**Affiliations:** From the Department of Clinical Neuroscience and Institute of Environmental Medicine (A.K.H., L.A.), and Department of Clinical Neuroscience and Center for Molecular Medicine (I.K., T.O.), Karolinska Institutet, Stockholm; Mathematical Statistics (O.H.), Stockholm University, Sweden; UCL/Farr Institute of Health Informatics Research (M.K.), London, UK; and Centre for Occupational and Environmental Medicine (L.A.), Stockholm County Council, Sweden.

## Abstract

**Objective:**

We hypothesize that different sources of lung irritation may contribute to elicit an immune reaction in the lungs and subsequently lead to multiple sclerosis (MS) in people with a genetic susceptibility to the disease. We aimed to investigate the influence of exposure to organic solvents on MS risk, and a potential interaction between organic solvents and MS risk human leukocyte antigen (HLA) genes.

**Methods:**

Using a Swedish population-based case-control study (2,042 incident cases of MS and 2,947 controls), participants with different genotypes, smoking habits, and exposures to organic solvents were compared regarding occurrence of MS, by calculating odds ratios with 95% confidence intervals using logistic regression. A potential interaction between exposure to organic solvents and MS risk HLA genes was evaluated by calculating the attributable proportion due to interaction.

**Results:**

Overall, exposure to organic solvents increased the risk of MS (odds ratio 1.5, 95% confidence interval 1.2–1.8, *p* = 0.0004). Among both ever and never smokers, an interaction between organic solvents, carriage of HLA-DRB1*15, and absence of HLA-A*02 was observed with regard to MS risk, similar to the previously reported gene-environment interaction involving the same MS risk HLA genes and smoke exposure.

**Conclusion:**

The mechanism linking both smoking and exposure to organic solvents to MS risk may involve lung inflammation with a proinflammatory profile. Their interaction with MS risk HLA genes argues for an action of these environmental factors on adaptive immunity, perhaps through activation of autoaggressive cells resident in the lungs subsequently attacking the CNS.

Multiple sclerosis (MS) is a chronic immune-mediated disease of the CNS in which the etiology involves both genetic and environmental factors. The strongest genetic associations with MS are located within the human leukocyte antigen (HLA) complex. The HLA-DRB1*15 allele provides an increased risk of MS with an odds ratio (OR) of approximately 3 whereas the HLA-A*02 allele of the HLA-A locus exerts a protective effect with an OR of approximately 0.5.^1,2^ Although predisposing gene variations have a role in disease development, previous estimations of the heritability may have been overestimated. Recently published population-based studies give concordance rates of approximately 17% for monozygotic twins as opposed to approximately 2% for dizygotic twins and siblings in general, compared to the general population in northern Europe with a prevalence of about 0.2%.^3^ These data strongly argue for an essential role of environmental exposures in determining disease risk, and these are important to define since they are potentially preventable.

Environmental factors influencing MS risk are Epstein-Barr virus infection,^4^ vitamin D status,^5^ sun exposure habits,^6^ adolescent obesity,^7,8^ and smoking.^9^ The recently described interaction between smoke exposure^10,11^ and MS risk HLA genes indicates that smoke exposure displays a considerably higher association with MS among participants with a genetic susceptibility to the disease.

A meta-analysis concluded that exposure to organic solvents, another source of lung irritation, is a risk factor for developing autoimmune diseases.^12^ Based on 15 studies, the OR of MS among participants exposed to organic solvents was 1.53 (95% confidence interval [CI] 1.03–2.29).

In a Swedish population-based case-control study, we aimed to investigate the influence of exposure to organic solvents on MS risk. We also explored a potential interaction between exposure to organic solvents and the HLA-DRB1*15 and HLA-A*02 alleles, taking smoking habits into consideration.

## Methods

### Study design and study participants

This study was based on Epidemiological Investigation of Multiple Sclerosis, which is a Swedish population-based case-control study comprising the general population aged 16 to 70 years. Newly diagnosed cases with MS were recruited via 40 neurology units, including all Swedish university hospitals. All cases were diagnosed by a neurologist according to the McDonald criteria. Two controls per case were randomly selected from the national population register (density sampling), frequency matched for the case's age in 5-year age strata, sex, and residential area. The study participants were recruited between April 2005 and December 2013. More detailed descriptions of study design and methods are given elsewhere.^13^

### Data collection

Information regarding environmental exposures and lifestyle factors was collected using a standardized questionnaire. Completed questionnaires were obtained from 2,325 cases and 4,948 controls; the response rate was 93% for cases and 73% for controls.

All participants were asked to provide a blood sample. Blood samples were available from 2,042 cases (88%) and 2,947 controls (59%) who answered the questionnaire.

### Standard protocol approvals, registrations, and patient consents

The study was approved by the regional ethical review board at Karolinska Institutet. All participants gave their informed consent to participate in the study.

### Data availability statement

Data related to the current article, intended for reasonable use, is in principle available from Lars Alfredsson, Karolinska Institutet. However, to be able to share data from the Swedish cohort, a data transfer agreement needs to be completed between Karolinska Institutet and the institution requesting to access the data. This is in accordance with new data protection legislation in Europe (GDPR [General Data Protection Regulation]). Anyone interested in obtaining access to the data from the Swedish cohort can contact Ingrid Kockum (ingrid.kockum@ki.se) to set up such a data transfer agreement.

### Definition of smoking habits and exposure to organic solvents

The initial clinical appearance of MS symptoms was used as an estimate of the disease onset, and the year in which this occurred was defined as the index year. The corresponding controls were given the same index year.

Participants were asked to provide information on occupational exposures by stating whether they had been exposed to a number of specified items including organic solvents, painting products, and varnish. For those exposed, participants were asked to specify during what period or periods they were exposed (calendar years) and how many hours per week they were exposed during each period. Participants who reported that they had been occupationally exposed to organic solvents, painting products, and/or varnish before the index year were defined as exposed to organic solvents.

Information on smoking was collected by asking detailed questions regarding current and previous smoking habits including duration of smoking and average number of cigarettes smoked per day. Smoking was only considered prior to the index year. Participants who had smoked before or during the index year were defined as ever smokers, whereas those who had never smoked before or during the index year were defined as never smokers.

### Genotyping and definition of genetic risk factors

All participants who donated blood were genotyped for the HLA-A and HLA-DRB1 alleles as previously described.^14^

The participants were classified according to carriage of any HLA-DRB1*15 allele vs no carriage. Absence of HLA-A*02 has a protective association to MS, and the participants were classified according to carriage of any HLA-A*02 allele vs no carriage.

### Statistical analysis

By means of logistic regression, the occurrence of MS in participants who reported exposure to organic solvents was compared with that of participants who had never been exposed, by calculating ORs with 95% CIs. Trend test for a dose-response relationship regarding exposure to organic solvents and risk of MS was performed by using a categorical variable for duration (5-year intervals) and cumulative dose (<52, 52–104, and >104 hours of exposure) in logistic regression models.

A possible interaction (super-additive associations) between carriage of HLA-DRB1*15, absence of HLA-A*02, and exposure to organic solvents, was evaluated by quantification of departure from additivity of effects. The overall interaction (i.e., due to all 2- and 3-way interactions) between HLA-DRB1*15, absence of HLA-A*02, and organic solvents with regard to MS risk was calculated by comparing the joint effect of the 3 risk factors to the situation when each one acts separately, using the total attributable proportion due to interaction (TotAP).^15^ Furthermore, we investigated a potential 2-way interaction between exposure to organic solvents and smoking in participants with different genotypes.

Both conditional and unconditional logistic regression was performed. However, only the results from the unmatched analyses are presented since these were in close accordance with those from the matched analyses but had higher precision in terms of more narrow CIs. All analyses were performed using Statistical Analysis System (SAS) version 9.4 (SAS Institute, Cary, NC).

### Confounding factors

All analyses were adjusted for age, sex, residential area, ancestry, and, when appropriate, smoking habits. Ancestry was dichotomized into Swedish vs non-Swedish origin. A participant who was born in Sweden, whose parents had not immigrated, was classified as Swedish. Smoking was dichotomized into ever or never smoking.

Other potential confounding factors adjusted for only had minor influence on the results and were not retained in the final analyses (appendix e-1, links.lww.com/WNL/A594).^16^

## Results

Our analysis of exposure to organic solvents and MS risk was based on 2,042 cases and 2,947 controls matched by age, sex, and residential area. The mean age at MS onset was 34 years and the median duration from the clinically overt disease onset to the diagnosis was 1.0 year. Almost all cases were recruited within 1 year after the diagnosis and the questionnaires were completed after a median of 2.0 years following the onset of the disease. Characteristics of cases and controls, by exposure to organic solvents, are presented in table 1.

**Table 1:**
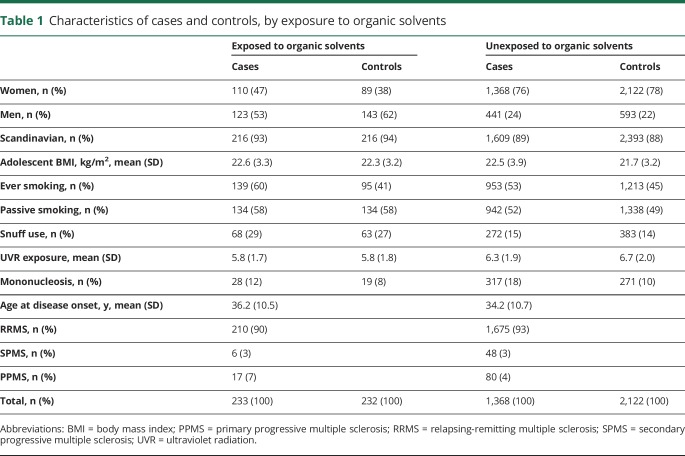
Characteristics of cases and controls, by exposure to organic solvents

A significant association was observed between exposure to organic solvents and increased risk of developing MS (adjusted OR 1.5, 95% CI 1.2–1.8, *p* = 0.0004). There were statistically significant trends showing increasing MS risk with increasing duration of exposure (*p* = 0.04) and total hours of exposure (*p* = 0.001). We calculated the ORs associated with different combinations of the genetic risk factors carriage of DRB1*15 and absence of HLA-A*02 in participants with different exposure to smoking and exposure to organic solvents, compared with unexposed participants without the genetic risk factors (table 2). Participants exposed to smoking and organic solvents carrying HLA-DRB1*15 and lacking HLA-A*02 had a 30-fold increased risk of developing MS compared with nonexposed participants without the genetic risk factors (OR 30.3, 95% CI 11.7–78.3) (table 2). The interactions among carriage of HLA-DRB1*15, absence of HLA-A*02, smoking habits, and exposure to organic solvents are illustrated in the figure.

**Table 2:**
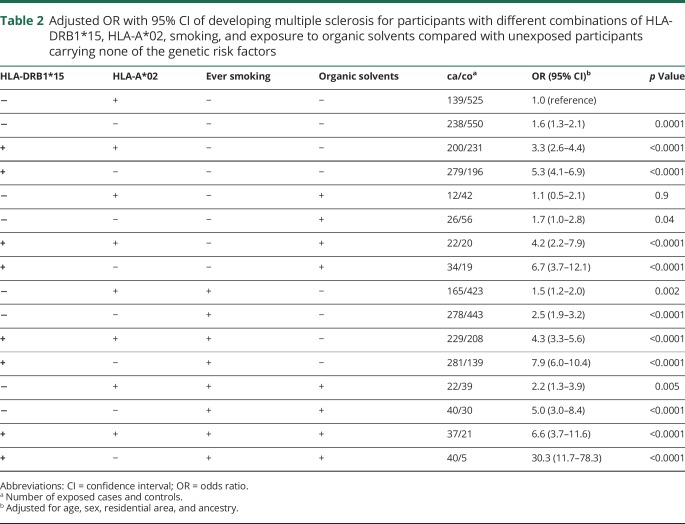
Adjusted OR with 95% CI of developing multiple sclerosis for participants with different combinations of HLA-DRB1*15, HLA-A*02, smoking, and exposure to organic solvents compared with unexposed participants carrying none of the genetic risk factors

**Figure:**
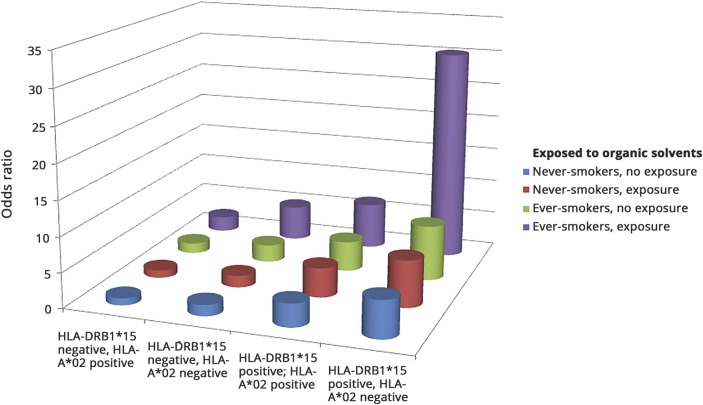
Odds ratios for different combinations of HLA-DRB1*15, HLA-A*02, smoking, and organic solvent status Odds ratios for different combinations of HLA-DRB1*15, HLA-A*02, smoking, and organic solvent status, compared with HLA-A*02 positive participants without the HLA-DRB1*15 allele and without the environmental risk factors. Based on data from table 2.

Overall, a significant interaction between exposure to organic solvents, carriage of HLA-DRB1*15, and absence of HLA-A*02 was observed with regard to MS risk (TotAP 0.6, 95% CI 0.3–0.8, after adjusting for the matching factors ancestry and smoking) (table 3). We also performed the analysis stratified by smoking, as displayed in appendices e-2 and e-3 (links.lww.com/WNL/A594). There were presence of interaction between organic solvents, carriage of HLA-DRB1*15, and absence of HLA-A*02 among both never smokers (TotAP 0.4, 95% CI 0.07–0.7) and smokers (TotAP 0.7, 95% CI 0.4–1.0).

**Table 3:**
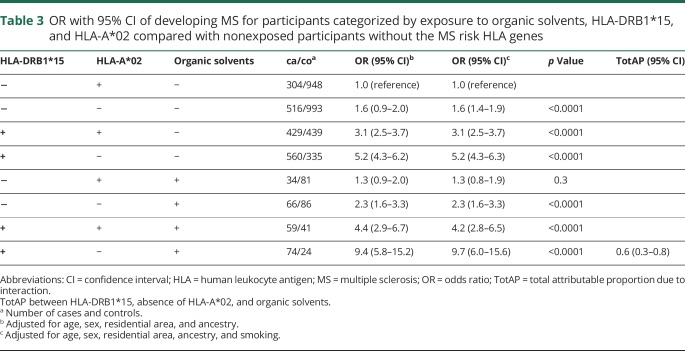
OR with 95% CI of developing MS for participants categorized by exposure to organic solvents, HLA-DRB1*15, and HLA-A*02 compared with nonexposed participants without the MS risk HLA genes

We then analyzed the interaction between organic solvents and smoking regarding MS risk in relation to different genetic contexts. Among participants with MS risk HLA genes, there was a significant interaction between exposure to organic solvents and smoking (attributable proportion due to interaction [AP] 0.4, 95% CI 0.2–0.7 among those with either of the genetic risk factors, and AP 0.7, 95% CI 0.4–1.0 among those with both genetic risk factors), whereas no significant interaction was observed among those without increased genetic risk (table 4).

**Table 4:**
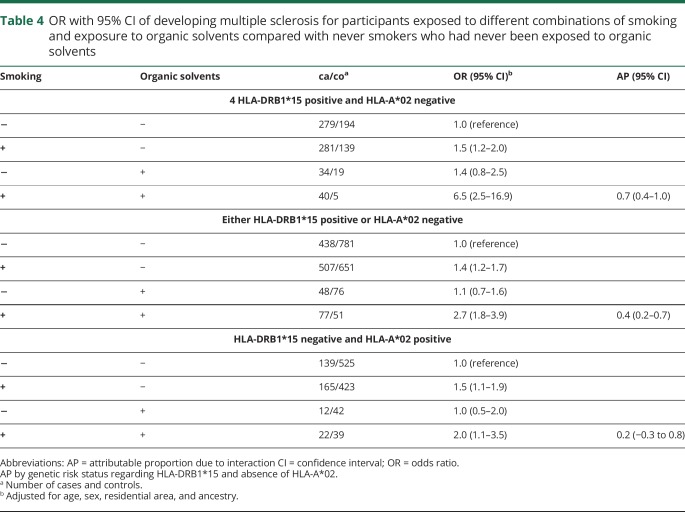
OR with 95% CI of developing multiple sclerosis for participants exposed to different combinations of smoking and exposure to organic solvents compared with never smokers who had never been exposed to organic solvents

All analyses were adjusted for age, sex, residential area, ancestry, and, when appropriate, smoking habits. The analyses were also adjusted for heredity, educational level, socioeconomic status, snuff use, body mass index at age 20, ultraviolet radiation exposure habits, supplemental vitamin D intake during the last 5 years, vitamin D status at inclusion, and a history of infectious mononucleosis. However, these factors had only a minor effect on the results and were not kept in the final analyses.

## Discussion

In our study, we calculated the association between exposure to organic solvents and MS risk, and demonstrated a significant interaction between HLA-DRB1*15, absence of HLA-A*02, and exposure to organic solvents, similar to the previously reported gene-environment interaction involving the same MS risk HLA genes and smoke exposure. Since the basic function of the HLA molecules is to present antigen to T cells, their interaction with smoking and organic solvent exposure strongly suggests that these environmental risk factors mechanistically act on the adaptive immunity.

The previous findings that smoking and exposure to passive smoking,^10,11^ but not oral tobacco,^17^ increase the risk of MS indicate that it is the lung irritation itself that drives the increased risk associated with smoke exposure. Smoking causes proinflammatory cell activation in the lungs and induces posttranslational modifications of proteins,^18–20^ which may be cross-reactive with CNS antigens. Furthermore, in experimental neuroinflammation, autoaggressive T cells have been shown to be present and available for triggering in the lungs, and after assuming migratory properties, they reach the CNS with ensuing inflammation.^21^

The interaction between lung irritative agents and HLA genes with regard to MS risk is consistent with class II allele-specific recognition of autoantigenic peptides in the lungs, resulting in organ-specific inflammatory disease depending on preferential peptide binding by allelic variants of class II molecules.

Expression of A*0201 molecules has been suggested to increase negative selection of CNS autoreactive T cells or modulate their autoreactivity.^22^ Absence of A*0201 may thus result in autoreactive T cells persisting and launching an immune response against the self-antigen.

Class I allele-specific suppression mediated by CD8^+^ cells has been demonstrated in experimental autoimmune encephalomyelitis.^23–25^ However, further research on CD8^+^ T cells in relation to allelic influences is strongly warranted.

The mechanism linking both smoking and exposure to organic solvents to MS risk may thus involve lung inflammation with a proinflammatory profile. We hypothesize that different sources of lung irritation may contribute to induce an immune reaction against modified self-proteins or against potentially autoaggressive cells resident in the lungs and promote MS development in people with a genetic susceptibility to the disease.

There is some evidence that environmental agents enter the CNS via the olfactory structures. Olfactory bulb and tract demyelination has been shown to be highly inflammatory and we cannot rule out the olfactory axis as a route by which organic solvents and smoking influence the initiation of the disease.^26,27^ The gut microbiota has emerged as a potential factor in MS development, and another mechanism by which smoking and organic solvents may affect MS risk is through microbiome alterations.^28,29^

The study was designed as a case-control study in which information regarding environmental exposures and smoking habits was collected retrospectively. We used incident cases of MS in order to minimize recall bias. The questionnaire contained a large number of questions regarding many potential environmental risk factors and no section in the questionnaire was given prime focus.

Organic solvents are liquid organic chemicals used to dissolve solid materials. It is difficult to analyze different solvents separately since most of the solvents are a mixture, and most people cannot specify to what specific agents they have been exposed. In this study, participants were defined as exposed to organic solvents if they reported previous exposure to organic solvents, painting products, and/or varnish. Among those who reported exposure to organic solvents, there were no differences regarding professions or line of work between cases and controls, indicating a similar reporting pattern. Furthermore, those who reported exposure to organic solvents stated occupational titles/activities that were consistent with those in which exposure to solvents is most highly probable.

A potential selection bias may arise during the recruitment process of cases and controls. Since the structure of the Swedish health care system provides equal free-of-charge access to medical services for all citizens, almost all cases of MS are referred to neurologic units, making them eligible to be part of the study. The response rate for participation in the study was 93% for cases and 73% for controls. Selection bias is probably modest because the prevalence of lifestyle factors, such as smoking, among the controls was consistent with that of the general population.^30^

Only controls who had responded to the questionnaire were asked to provide a blood sample. There were no significant differences in age, sex, smoking habits, or exposure to organic solvents between those who provided blood and those who did not, indicating that selection bias did not take place in this step. It seems unlikely that our findings would be affected by bias to a large extent, especially since such a bias would depend on HLA types.

A larger dataset than ours would be needed to investigate the interaction between organic solvents, smoking, and both genetic risk factors, among women and men, respectively. However, a significant interaction between the genetic risk factors and organic solvents was observed among both sexes when the exposure exceeded 1 hour per week during at least 1 year (AP 0.6, 95% CI 0.01–1.2 among women, and AP 0.6, 95% CI 0.04–1.1 among men). Furthermore, the interaction between smoking and organic solvents among participants with both genetic risk factors was similar among women and men (AP 0.7, 95% CI 0.2–1.3 among women and AP 0.7, 95% CI 0.2–1.1 among men), whereas no significant interaction was observed among those without genetic risk factors (AP 0.1, 95% CI 0.8–1.0 among women and AP 0.2, 95% CI 0.6–1.0 among men).

In our study, we calculated the overall interaction (i.e., due to all 2- and 3-way interactions) between HLA-DRB1*15, absence of HLA-A*02, and organic solvents with regard to MS risk, by comparing the joint effect of the 3 risk factors to the situation when each one acts separately, using the TotAP. The observed TotAP was 0.6 (95% CI 0.3–0.8) after adjusting for the matching factors, ancestry, and smoking (table 3). Part of this interaction consists of the 2-way interaction between HLA-DRB1*15 and absence of HLA-A*02. For comparison, the AP for the 2-way interaction between these 2 genetic risk factors was 0.3 (95% CI 0.2–0.4).

We demonstrate a significant interaction between the MS risk HLA genes and exposure to organic solvents regarding MS risk, similar to the previously reported gene-environment interaction involving the same MS risk HLA genes and smoke exposure. We hypothesize that different sources of lung irritation may contribute to induce an immune reaction against modified self-proteins induced by lung irritation or against potentially autoaggressive cells resident in the lungs, and promote MS development in people with a genetic susceptibility to the disease.

## Glossary

AP: attributable proportion due to interaction
CI: confidence interval
HLA: human leukocyte antigen
MS: multiple sclerosis
OR: odds ratio
TotAP: total attributable proportion due to interaction
